# Supplementary material for: Causal interplay between lactose intolerance and gut microbiota: a combined bidirectional Mendelian randomization and in vivo validation study
Source: Front Nutr. 2026 Jun 1;13:1803337. doi: 10.3389/fnut.2026.1803337 (PMC13265576; doi:10.3389/fnut.2026.1803337)
Supplement: Supplementary file 1 [file Data_Sheet_1.zip › supplementary materials/Forward/leave one out cross validation/ebi-a-GCST90027523.finngen_R12_E4_LACTONAS.pdf]

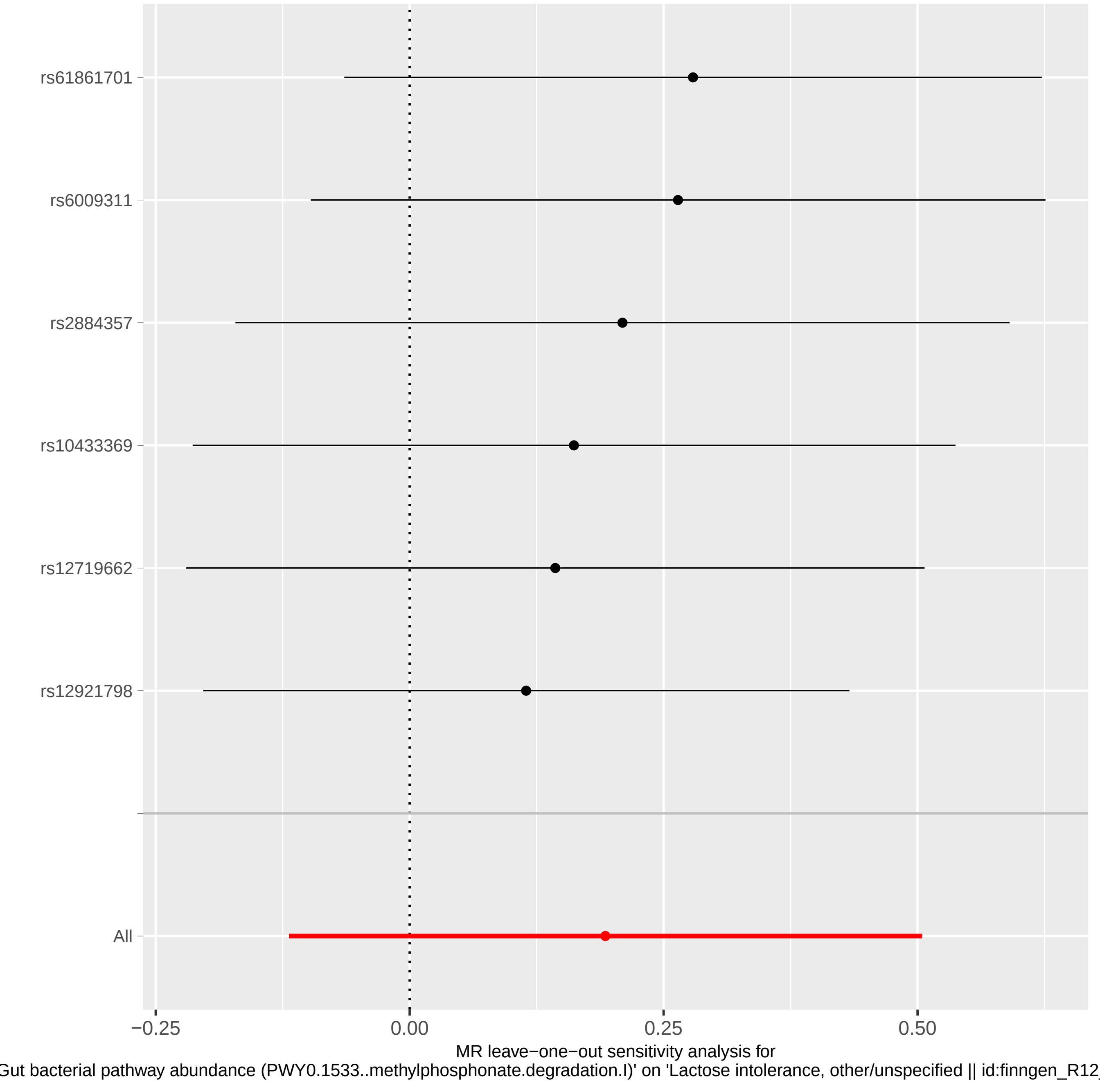
rs61861701
rs6009311
rs2884357
rs10433369
rs12719662
rs12921798
All
−0.25
0.00
0.25
0.50
MR leave−one−out sensitivity analysis for
Gut bacterial pathway abundance (PWY0.1533..methylphosphonate.degradation.I)' on 'Lactose intolerance, other/unspecified || id:finngen_R12_
